# Comparison of gut microbiota diversity between wild and captive bharals (*Pseudois nayaur*)

**DOI:** 10.1186/s12917-019-1993-7

**Published:** 2019-07-12

**Authors:** Xiangwen Chi, Hongmei Gao, Guosheng Wu, Wen Qin, Pengfei Song, Lei Wang, Jiarui Chen, Zhenyuan Cai, Tongzuo Zhang

**Affiliations:** 1 Key Laboratory of Adaptation and Evolution of Plateau Biota, Northwest Institute of Plateau Biology, Chinese Academy of Sciences, No.23 Xinning road, Chengxi district, Xining, TN 810008 Qinghai China; 2 University of Chinese Academy of Sciences, No.19 Yuquan road, Shijingshan district, Beijing, TN 100049 China; 3 Qinghai Provincial Key Laboratory of Animal Ecological Genomics, No.23 Xinning road, Chengxi district, Xining, TN 810008 Qinghai China; 4 Qinghai-Tibet Plateau Wildlife Zoo, No.9 Xingzhi road, Chengxi district, Xining, TN 810008 Qinghai China

**Keywords:** Bharal (*Pseudois nayaur*), 16S rRNA gene, Gut microbiota, Health assessment, High-throughput sequence analysis

## Abstract

**Background:**

Gastrointestinal microbiota play an important role in animal host immunity, nutrient metabolism, and energy acquisition, and have therefore drawn increasing attentions. This study compared the diversity of the gut microbiota of both wild and captive bharals, which is an ungulate herbivore of caprid from the Qinghai-Tibet plateau.

**Results:**

The sequencing of the V4-V5 region of the 16S rRNA gene via high-throughput sequencing technology showed that the dominant bacterial phyla are Firmicutes and Bacteroides both in wild and captive bharals. However, their abundance differed significantly between groups. Firmicutes were significantly higher in wild bharals, while Bacteroides were significantly higher in captive bharals. Different diets are likely a key influencing factor in the diversity and abundance of gut microbiota in bharals.

**Conclusions:**

Changes in diets affect the diversity of gut microbiota and the relative abundance of pathogenic bacteria, increasing the risk of diseases outbreak in captive bharals. The results of this study suggest that the structure and function of the gut microbiota should be regulated via dietary intervention, accurate provision of an individualized diet, and optimization of the functional network of gut microbiota and its interaction with the host. This will improve the ex situ protection of wild animals.

## Background

Due to the use of modern molecular technology, the gut microbiota can be utilized as a signal hub that combines environmental inputs (e.g., diet) with genetic and immune signals. All of this affect the host metabolism, immunity, and infection responses [1], and play an important role in the development of the immune system and in animal health [2–6]. The diversity and abundance of the host intestinal flora are influenced by factors such as species [7], food [8–10], genotype, and age of the host [11]. Further research indicated that changes in the dietary patterns of the host can lead to rapid changes in the structure of the microbial community [12], which in turn exerts a profound impact on the health of the host [13]. The role of the diet in the regulation of the composition and metabolic activities of the gut microbiota has been increasingly recognized. Furthermore, the relationship between gut microbiota and animal health has been extensively studied.

The bharal (*Pseudois nayaur*) is a member of the Artiodactyla, Bovidae, Caprinae, Pseudois, and a national second-class protected animal on the list of key wildlife protection in China [14]. The bharal is mainly distributed throughout the Qinghai-Tibet Plateau and its surrounding areas, including Tibet, Yunnan, Sichuan, Xinjiang, Qinghai, Gansu, Inner Mongolia, Ningxia and Shaanxi [14]. It is one of the large hoofed animals in China and presents the most widely distributed cloven hoofed animal with the largest number on the Qinghai-Tibet Plateau [15, 16]. The major predator of wild bharals is the snow leopard (*Uncia uncia*) [17, 18], and this predation is important to maintain both the stability of ecosystems and species diversity [15]. At present, research on bharals focuses on population ecology [14, 19], behavioral ecology [20], and system evolution [21, 22]. During winter, bharals often face food shortage, which even lead to death [14]. Therefore, it is very important to study the digestibility and utilization rate of food in bharals. Its gut microbiota exerts a very important influence on this function. Artificial captive protection is an effective means of protection; however, it remains unclear whether the health status of captive bharals will be affected when animals are faced with novel living conditions. Therefore, it is necessary to study the relationship between changes in the gut microbiota and animal health.

This study investigated the composition and structure of the gut microbiota of bharals. The results lead to a better understanding of the digestive mechanism, provide a theoretical basis for the monitoring of abnormal physiological status, control the occurrence of diseases, and optimize the energy conversion rate of the food. The findings provide an important theoretical basis for the research digestive physiology of captive bharals. The results provide information to improve the diet of animals as well as for diagnosis and treatment of intestinal diseases. Furthermore, new research directions are initiated for the development of intestinal microecological agents.

## Results

### Sequencing data

A total of 8,217,442 high-quality reads were obtained after data quality control, and fecal samples were classified into 3,878 OTUs (operational taxonomic units), 2,443 of which were in the captive group and 3,166 in the wild group.

**Fig. 1:**
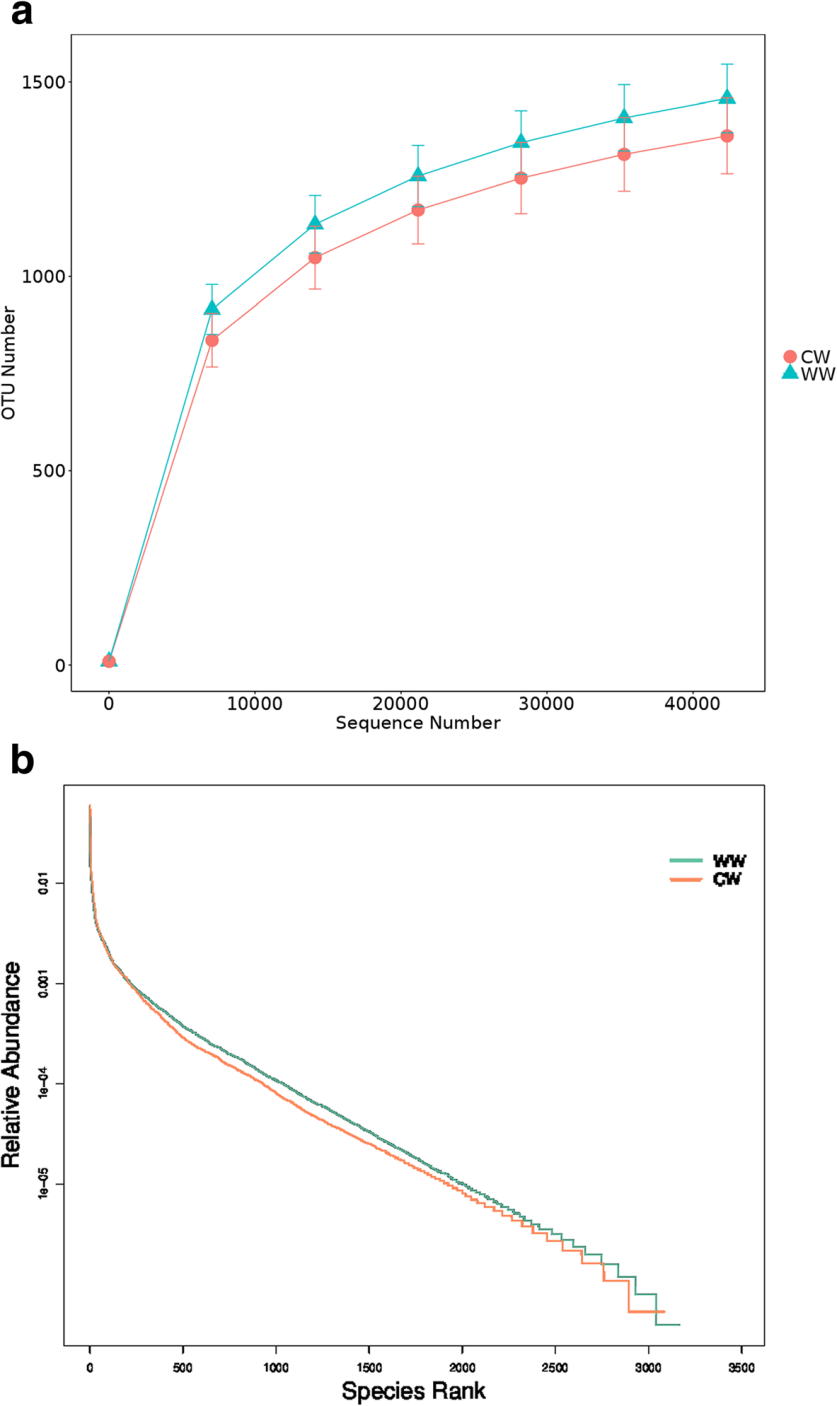
Rarefaction curves **(a)** and rank abundance curves **(b)**. CW for captive group, WW for wild group

Rarefaction curves and rank abundance curves are commonly used to describe the diversity of samples within a group. The rarefaction curves directly reflect the rationality of the sequencing data volume, and indirectly reflect the species richness of samples, as shown in Fig. 1a. Since the curves are smooth, a higher data volume would only yield a low number of OTUs, indicating that the volume of sequencing data is sufficiently reasonable. Rank abundance curves provide a visual representation of species richness and sample uniformity. In the horizontal direction, a greater span of the curve indicates a higher species richness, while in the vertical direction, a smoother curve indicates a more homogeneous species distribution [23] (Fig. 1b).

### Bacteria composition and relative abundance

**Fig. 2:**
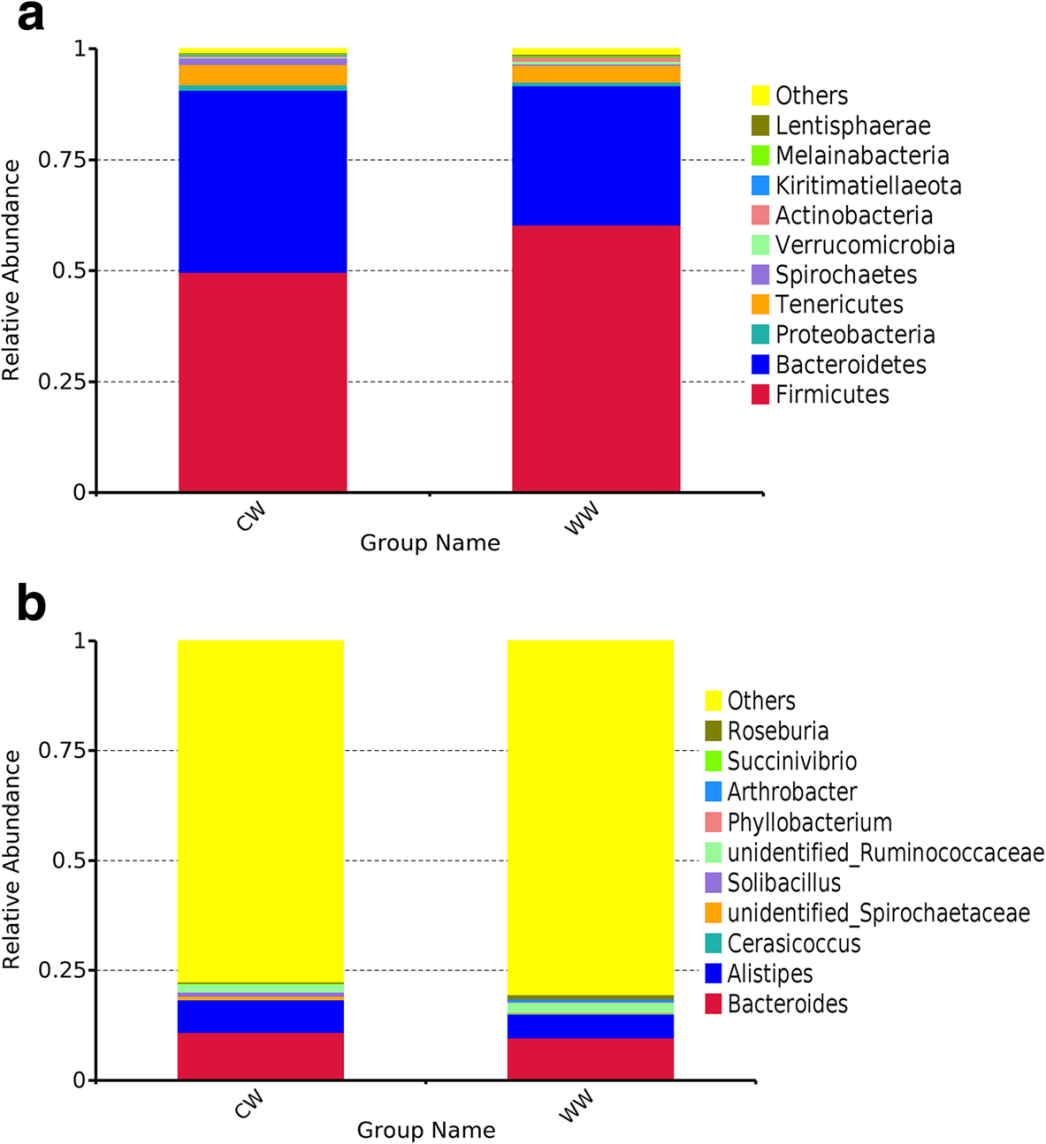
Relative abundance histogram. Fecal microbial composition of wild (WW) and captive (CW) bharals at the phylum **(a)** and genus **(b)** level. Each bar represents the top ten bacterial species ranked by the relative abundance in each group

In order to show the relative abundance of bacteria communities more intuitively, we selected the top 10 taxa and generated the relative abundance superposition histogram at phylum and genus level respectively in Fig. 2.

At the phylum level, Firmicutes and Bacteroidetes are the dominant phyla of both wild and captive groups; however, the abundance of Firmicutes (60.35%) in wild bharals was significantly higher (*P* < 0.01) than that of captive bharals (49.68%). The abundance of Bacteroides (40.98%) was significantly higher (*P* < 0.01) in captive bharals than in wild bharals (31.35%). In wild and captive groups, the main genera are Bacteroides and Alistipes, and the abundances of Bacteroides and Alistipes in captive bharals were significantly higher (*P* < 0.05) than that in wild bharals.

### Analysis of discrepancies between groups

The goods coverage index exceeded 99%, indicating a high level of diversity coverage in the samples. The observed species, Shannon index, and Simpson index in the wild group were significantly higher than in the captive group (*P*_Observed species_ < 0.01, *P*_Shannon_ < 0.01, and *P*_Simpson_ < 0.01).

To further analyze whether significant differences affected the microbiome structure between the wild and captive group, the multi response permutation procedure (MRPP) significance analysis method was used. The result was A = 0.1153 > 0, indicating that differences between groups were higher than within groups; consequently, the applied study grouping was reasonable and a significant difference (*P* = 0.001) was found between wild and captive groups.

**Fig. 3:**
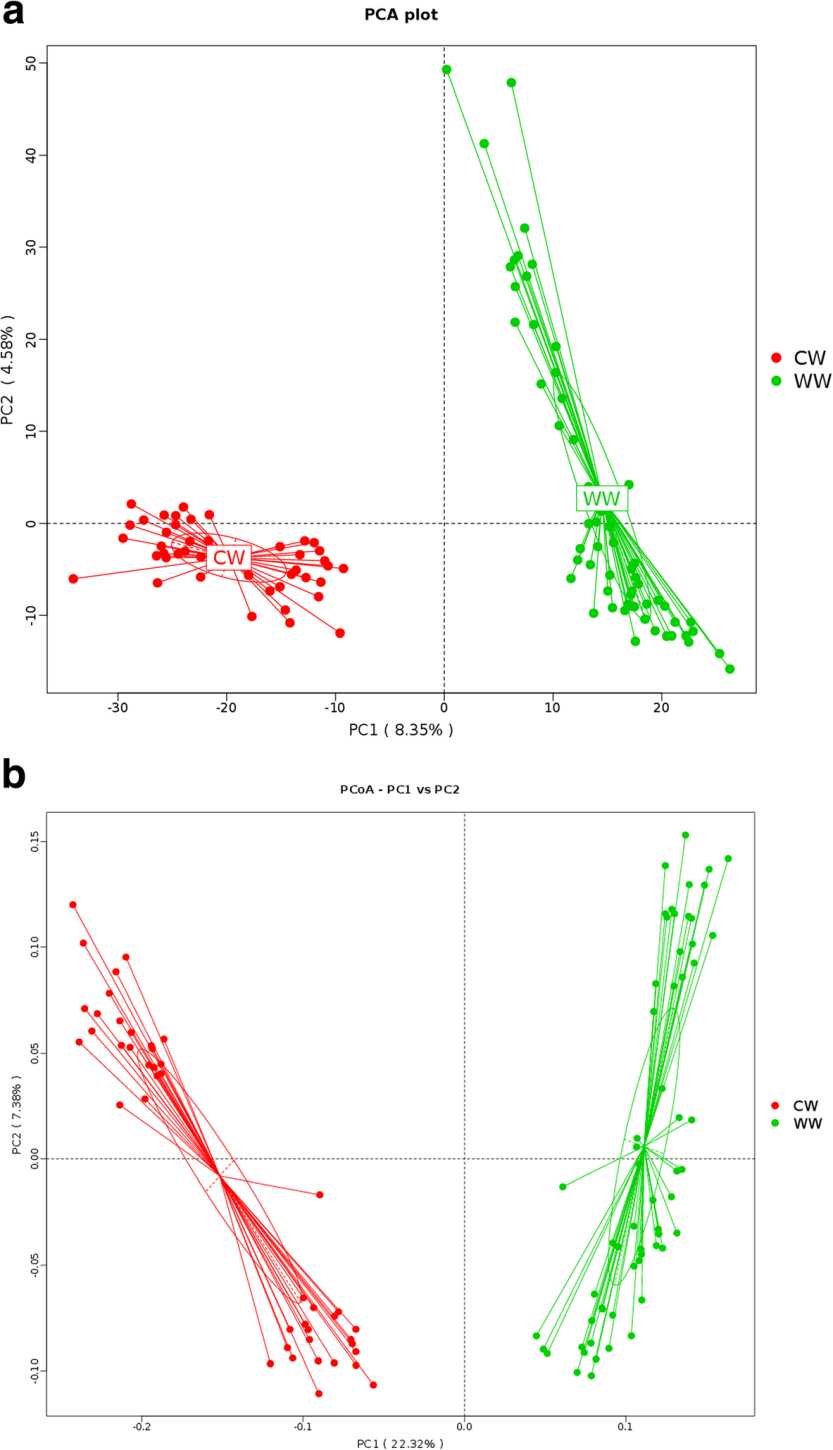
PCA **(a)** and PCoA **(b)** plot of the bacterial population structures. The green and red dots represented wild (WW) and captive (CW) bharals samples respectively

Furthermore, to assess the differences between wild and captive groups, principal component analysis (PCA) (Fig. 3a) and principal coordinates analysis (PCoA) (Fig. 3b) were used. The distance between the points in all graphs reflects the degree of similarity of their respective microbial flora structures. All graphs reflect the obvious difference of samples in each group.

**Fig. 4:**
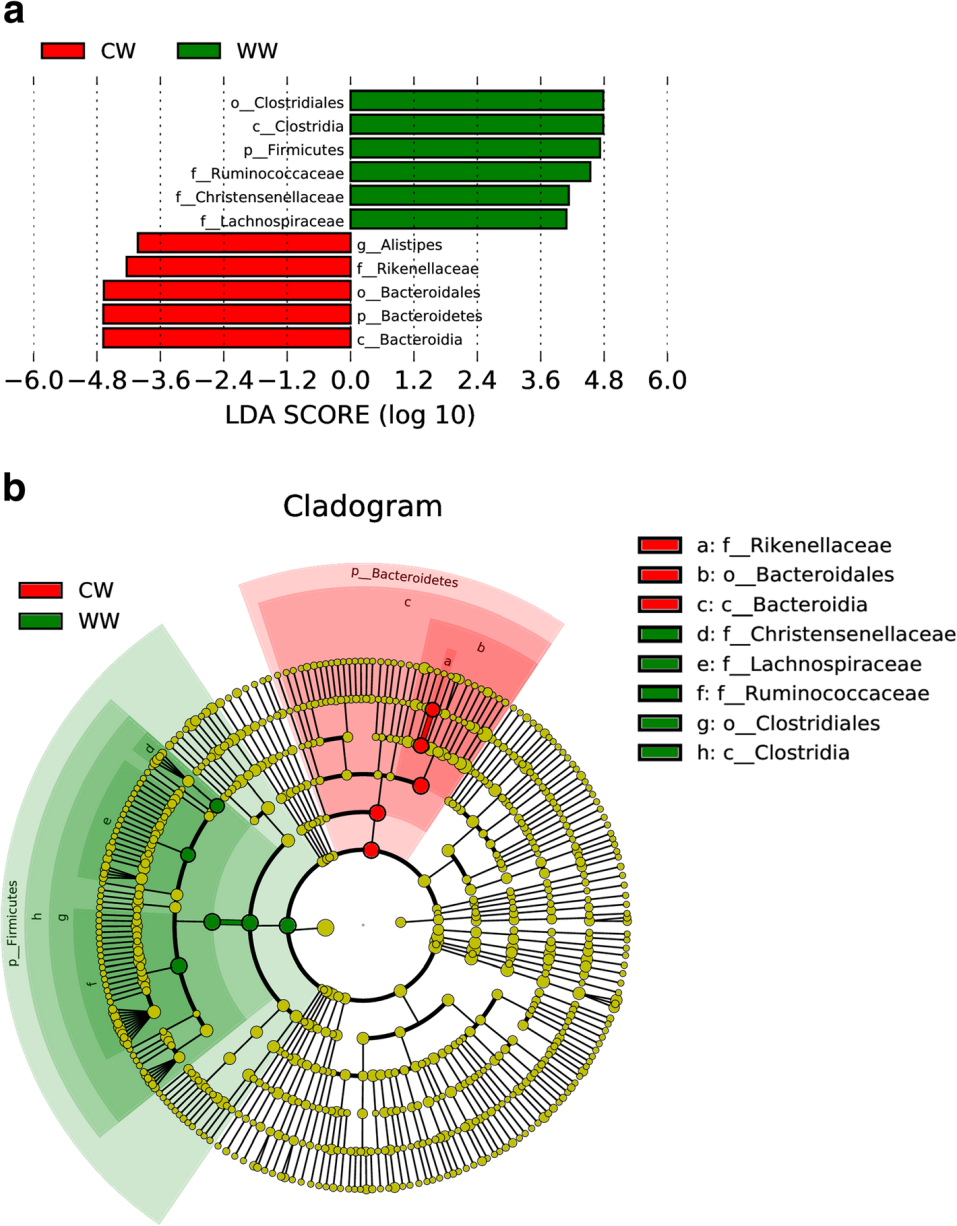
The results of LEfSe (LDA Effect Size) analysis. The histogram of LDA score**(a)** showed the biomarkers with significant differences between groups. The length of the column (LDA Score) represents the influencing degree of biomarkers, In the cladogram**(b)**, the circle radiated inside-out represented the classification of phylum to genus level. Each small circle at different classification levels represented a taxon and the diameter of small circle is proportional to the relative abundance. The species not with significant differences were colored by yellow and biomarkers were colored by different groups. CW for captive group, WW for wild group

Using the LDA (local-density approximation) Effect Size (LEfSe) analysis method, species with significant differences between groups were selected. The results included three parts: a LDA value distribution histogram, an evolutionary branch diagram (phylogenetic distribution), and an abundance comparison diagram of biomarkers with statistical differences (LDA SCORE > 4) between groups (Fig. 4). According to the LDA scores, biomarkers with statistically significant differences between both groups are listed. The microbial communities that play an important role in each group were identified with the evolutionary branch diagram. In the wild group, Firmicutes, Clostridia, Clostridiales, Ruminococcaceae, Christensenellaceae and Lachnospiraceae are very important. In the captive group, important species are Bacteroidetes, Bacteroidia, Bacteroidales, Rikenellaceae and Alistipes.

## Discussion

At the phylum level, the core microflora of the gut microbiota consisted of Firmicutes and Bacteroides in both captive and wild bharals, accounting for more than 90% of the total gut microbiota. This result coincided with previous studies of the gut microbiota in other ruminants [24–27]. However, their abundances showed significant differences (*P* < 0.01) between groups. In ruminants, Firmicutes play an important role in fiber and cellulose degradation, and can degrade cellulose into volatile fatty acids, which can be used by the host [28]. Firmicutes and Bacteroidetes increase with increasing hay content in the food, and this characteristic of Firmicutes is particularly prominent [29]. Although wild bharals have access to a wide variety of food sources, due to the harshness of the environment in winter, they mainly feed on grasses with high fibre content. Compared to captive bharals, wild bharals showed a higher abundance of Firmicutes, which leads to improved digestion and absorption of nutrients. Bacteroides can promote digestion, decompose polysaccharides and proteins, improve the utilization rate of assimilated nutrients [30, 31], and maintain the balance of intestinal microecological system [32]. The diet of captive bharals consists of semi-dried oat grass (*Arrhenatherum elatius*), carrot (*Daucus carota*), and artificial fodder. The utilized recipes are simple in structure; however, the protein, polysaccharide, and fat contents of the artificial fodder are relatively high. This might be the reason for the higher abundance of Bacteroides in the intestinal flora of captive bharals compared to wild bharals.

LEfSe analysis showed a number of bacteria that play an important role in metabolism of nutrients and the health of the host. Previous studies have reported that Alistipes shows a correlation with metabolites such as short-chain fatty acids, oligosaccharides, and amino acids. Lachnospiraceae and Ruminococcaceae belong to butyrate-producing bacteria. These bacteria and metabolites exert an important impact on host health [33, 34].

In addition, the Alpha-diversity of the captive group was significantly lower than that of the wild group. The food sources accessible to wild animals are more diverse, thus providing bharals with many different types of nutrition, which may require a more diversified gut microbiota to help bharals complete digestion and utilization of these nutrients [35, 36].

Further research has shown that many chronic diseases are caused by a decrease in the diversity of gut microbiota, including inflammatory gut disease and diabetes [37]. Comparison of the inter-group differences of the intestinal flora showed that the relative abundances of Spirochaetes (*P* < 0.01), Acidobacteria (*P* < 0.01) and Gemmatimonadetes (*P* < 0.01) in the captive group were significantly higher than in the wild group; these bacteria showed a certain pathogenicity [38, 39]. However, Ruminococcaceae, Lachnospiraceae, and Christensenellaceae in the captive group were significantly lower than in the wild group; these bacteria are beneficial microbiota in the host intestines [40]. These results suggest that captive bharals may face a higher risk to catch diseases than wild bharals.

This relationship between gut microbiota and host health, and in particular, the impact of microbial dissonance on host health, indicates the importance of feasible strategies to optimize the gut microbiota via diet. This means that diseases caused by changes in the gut microbiome can be regulated through the diet of the host. Food types can be reasonably chosen, a number of nutrients can be appropriately supplemented, and the healthy development of gut microbiota can be promoted. This will be conducive to the protection of wildlife ex situ.

## Conclusion

This study described and compared the gut microbiota of wild and captive bharals and significant differences were found between both groups. The difference in diet may have caused a decrease of gut microbiotic diversity and an increase of relative abundance of pathogenic bacteria. The result would be an increased susceptibility to diseases in captive bharals, which is extremely unfavorable for their protection. These findings provide further research directions for the study of the effect of gut microbiota on the growth and development of bharals. Moreover, this study provides theoretical guidance for the diagnosis and detection of intestinal diseases in bharals, and can also be used as a reasonable and balanced reference for the diet of captive bharals. The findings provide a theoretical basis for the screening of probiotics and development of intestinal microecological agents.

## Methods

### Feces samples collection

The difficulties of wildlife sampling and the religious beliefs of ethnic minorities are considered, feces samples were selected as test material in this study. At present, most of the research on wildlife in this aspect takes fecal samples as research objects, the results describe the structure of the gut microbiota of bharals. Therefore, during January 2018, a total of 240 fresh feces samples of wild bharals were collected from different regions near the Donggeicuona Lake in Maduo County, Qing Hai, China. The average temperature of Maduo in January is − 16.8 °C, which retained the freshness of the feces of wild bharals as much as possible. The collected samples were temporarily stored in an in-vehicle refrigerator (− 20 °C).

Forty-four fresh feces samples were collected from 11 bharals in captivity at the Qinghai-Tibet Plateau Wildlife Zoo during December 2017. To prevent sample contamination, the bharals enclosure was cleaned in advance and none of the sample donors received antibiotic or probiotic therapy for the past three months. The collected samples were temporarily stored in dry ice (− 50 °C). All feces samples were eventually frozen and stored at − 80 °C for further analyses.

### DNA extraction

Total genome DNA of samples was extracted by CTAB method using QIAamp^®^ Stool Mini Kit (Qiagen, Germany). DNA concentration and purity were detected with 1% agarose gel. Diluted the DNA to 1 ng/μL with sterile water.

### PCR amplification, purification and sequencing

16S rRNA genes of distinct regions (V4-V5) were amplified used specific primer (515F:5′-GTGCCAGCMGCCGCGGTAA-3′; 907R:5′-CCGTCAATTCCTTTGAGTTT-3′) with the barcode [41]. All PCR reactions were 30 μL systems, including 15 μL Phusion^®^ High-Fidelity PCR Master Mix (New England Biolabs), 3 μL (2 μM) forward and reverse primers, 10 μL (10 ng) template DNA and 2 μL ddH_2_O. The thermal cycling consisted of pre-denatured at 98 °C for 1 min, followed by 30 cycles, including denaturation at 98 °C for 10 s, annealing at 50 °C for 30 s, and extension at 72 °C for 30 s. Finally, 72 °C for 5 min.

The 1 × loading buffer (contained SYB green) of the same volume was mixed with the PCR products and detected by 2% agarose gel electrophoresis. PCR products was mixed in equidensity ratios. Then, mixture PCR products was purified with GeneJET™ Gel Extraction Kit (Thermo Scientific).

Sequencing libraries were generated using Ion Plus Fragment Library Kit 48 rxns (Thermo Scientific). The library was sequenced on an Ion S5™ XL platform to produce 400 bp/600 bp single-end reads.

### Sequence processing and statistical analysis

Get single-end reads and filtered the quality of the raw reads to obtain high-quality clean reads [42] (V1.9.1, http://cutadapt.readthedocs.io/en/stable/). By using UCHIME algorithm (http://www.drive5.com/usearch/manual/uchime_algo.html) [43], the reads were compared with the reference database to detect chimera sequences, and then removed [44]. Then the Clean Reads finally obtained. Sequences analysis were performed by Uparse software (Uparse v7.0.1001, http://drive5.com/uparse/) [45]. Sequences with ≥97% similarity were assigned to the same operational taxonomic units (OTUs). Representative sequence for each OTU was screened for further annotation [41, 46].

Qiime software (Version 1.9.1) was used to analyze the differences of alpha diversity indexes between groups. R software (Version 2.15.3) was used to plot rarefaction curves, rank abundance curves, and stacked histograms of the relative abundance. Ade4 and ggplot2 packages in R were used for PCA and PCoA analyses, respectively. LEfSe software (LEfSe 1.0) was used for LEfSe analysis, which defaulted to a filter value of 4 for the LDA Score. For MRPP analysis, the MRPP function of the vegan package for R was used.

## Data Availability

The datasets generated and analysed during the current study are not publicly available due to further analysis at later stage, but are available from the corresponding author on reasonable request.

## Abbreviations

LEfSe: Linear discriminant analysis coupled with effect size
MRPP: Multi Response Permutation Procedure
OTU: Operational taxonomic unit
PCA: Principal component analysis
PCoA: Principal co-ordinate analysis
PCR: Polymerase chain reaction
rRNA: Ribosomal ribonucleic acid
